# Prevalence and Trends in Obesity among China’s Children and Adolescents, 1985–2010

**DOI:** 10.1371/journal.pone.0105469

**Published:** 2014-08-20

**Authors:** Hongpeng Sun, Yana Ma, Di Han, Chen-Wei Pan, Yong Xu

**Affiliations:** Department of Child Health, School of Public Health, Soochow University, Suzhou, China; INIA, Spain

## Abstract

**Objectives:**

We examined the prevalence of and trends in obesity among children and adolescents in China (1985–2010).

**Methods:**

We used data from the 1985, 1991, 1995, 2000, 2005, and 2010 Chinese National Surveys on Students’ Constitution and Health (CNSSCH). The CNSSCH is a national survey of physical fitness and health status in Chinese students that uses multistage stratified sampling of 31 provinces and municipalities. A subject was considered obese or overweight if weight-for-height exceeded the 20% or 10% of standard weight-for-height. The standard weight-for-height was the 80th percentile for sex- and age-specific growth charts.

**Results:**

The age-adjusted prevalence of obesity and of overweight and obesity combined was 8.1% (95% CI, 8.0–8.3%) and 19.2% (95% CI, 19.1–19.4%) among children and adolescents 7–18 years in age. Obesity was more likely to be present among children or adolescents who were male (RR, 1.93; 95% CI, 1.90–1.97), urban (RR, 1.99; 95% CI, 1.95–2.02), or 10–12 years (RR, 1.43; 95% CI, 1.40–1.46). Trend analyses of the 25-year period revealed a significant increasing trend in males (RR, 1.59; 95% CI, 1.58–1.60) and in females (RR, 1.49; 95% CI, 1.48–1.50). The rate of increase in obese or overweight prevalence was highest in boys from rural areas (9% annual increase).

**Conclusions:**

During 1985–2010, there was a significant and continuous increase in the prevalence of obesity in children and adolescents. Obesity is epidemic in China, but may be reduced with evidence-based interventions (e.g., school intervention programs).

## Introduction

Obesity is a major risk factor for cerebrovascular disease, hypertension and diabetes, and the prevalence of obesity is increasing in China. Children who are overweight are more likely to become overweight or obese adults [1]. Childhood obesity is linked to serious complications in adulthood that include an associated increased risk of ill health and premature death [2]. In addition to an increased risk for future disease, being overweight or obese during childhood has important short-term metabolic effects. These conditions adversely affect growth rate, blood pressure, and lipid and glucose levels. They also increase a child’s risk for other health-related complications, including respiratory and musculoskeletal conditions [3]. The negative psychosocial consequences of overweight and obesity are also important. Obese children have an increased sense of loneliness, sadness, and nervousness, and are more likely to engage in risk-taking behaviors such as tobacco and alcohol consumption [4].

The prevalence of childhood obesity in China has gradually increased to the point where it is now similar to developed countries [5], [6]. Improvements in the economy and the ‘modernization’ of society have led to a continuous increase in obesity rates in preschool and school children over the past decade [7]–[9]. It has been reported that the changing pace of obesity prevalence in urban Chinese children [10]. However, data related to the rural areas of China are currently unknown. Moreover, logistic regression to estimate the prevalence odds ratio (OR) is often used to assess the gender and age differences and trends of obesity, and the odds ratio always overstates the relative risk [11]. Rate ratio (RR) that can estimate relative risk is an expected target.

The Chinese National Survey on Students’ Constitution and Health (CNSSCH) is used to monitor national trends in the health of students. Since 1985, CNSSCH height and weight data have been used to estimate obesity levels among Chinese children and adolescents. Data from these surveys have provided a broad-based indication of the trends confronted by China. The most recent prevalence of overweight and obesity values can be obtained from the height and weight data collected in 2010. The objectives of this study were to analyze the most recent estimates of overweight and obesity prevalence, and to estimate the variation among different demographic and socioeconomic groups in China’s school-age children and adolescents for 2010. We also evaluated trends in overweight or obesity prevalence among children and adolescents aged 7 through 18 years between 1985 and 2010.

## Subjects and Methods

Data were obtained from the CNSSCH, which is a series of complex multistage, cross-sectional, nationwide surveys that use standardized methodology. In 1985, CNSSCH became a continuous survey and data have been released every 5 years. The sampling methods were the same in all CNSSCH at different time points. All subjects were primary and secondary school students aged 7–18 years randomly selected from 31 mainland provinces. In each of the provinces, the subjects were classified by gender and region (urban or rural), and each of the four groups had an equal size of sample from three socioeconomic classes (upper, middle, and lower). Then three urban and three rural residential areas were selected, respectively. Several primary and secondary schools were randomly selected from a list compiled by each area’s Educational Committee. A list of students from grades 1–12 was compiled, and a random selection of two or three classes (depending on their size) was made within each grade level. This report is based on survey data that were collected in 1985 (*n* = 409,946), 1991 (*n* = 175,555), 1995 (*n* = 208,638), 2000 (*n* = 223,772), 2005 (*n* = 234,153), and 2010 (*n* = 215,203). All of the subjects were Han Chinese, who comprise 92.7% of the nationwide population.

The surveys included a standardized physical examination, which were conducted in a mobile examination center. The protocols that were followed to select the sample and conduct the interviews and examinations were similar to the protocols used for previous surveys [5], [6], [12]. Ethical approval was obtained from Peking University’s Medical Research Ethics Committee. The CNSSCH is a government’s welfare project which aims to promote the growth of children and adolescents. Verbal informed consent was obtained from each participant and their parents. They were very willing to participate in the project to obtain physical examination. Students or parents can refuse to participate in this project, and were informed that their information would be kept confidential. And our study analyzed the data anonymously. The ethics committees approved this consent procedure. To ensure the validity of comparisons among samples, the subjects in each group were randomly selected at various time periods. Detailed descriptions of these surveys have been published [5], [6], [12].

Physical examinations included measurement and recording of individuals’ height and weight. These anthropometric measurements were conducted by well-trained health workers who followed a reference protocol. Weight was measured to the nearest 0.10 kg with a balance-beam scale while the subjects were wearing lightweight clothing. Height was measured to the nearest 0.10 cm with a portable stadiometer while the subjects were barefoot. Weight category among children and adolescents was defined based on a standardized weight-for-height from the sex- and age-specific 80th percentile weight on the same height population [13]. The standards were created from the 1985 CNSSCH data (237476 males and 233639 females, aged 7–22 years). Overweight was defined, by age and sex, as 110–119.9% of standard weight-for-height. Obesity was defined as ≥120% of standard weight-for-height.

Age-adjusted prevalences of six periods were adjusted by the direct method to the 2010 China’s Census population using the age groups (7–9 years, 10–12 years, 13–15 years, and 16–18 years). The prevalence of overweight and obesity in 2010 were presented by sex, age, and area (urban and rural) subgroups. Univariate methods were not used to estimate P-values for differences by sex, age group, area, and survey, because high statistical power was achieved from the large sample sizes. The prevalence of overweight and obesity for sex-specific age groups were plotted for six survey periods (1985, 1991, 1995, 2000, 2005, and 2010) over a 25-year period.

To test trends in prevalence of overweight or obesity for children and adolescents during 1985–2010, we used generalized linear models (GLM) with a log function based on the binomial distribution [11], [14]. GLM was also used to generate rate ratios (RRs) between survey periods, adjusted for age group, sex, and area. Trends in overweight or obesity were also analyzed in specific GLM models stratified by sex, age group, and/or area. Linear trends in the prevalence of obesity over the six survey periods overall and by urban/rural were assessed with sex-specific GLM models with survey period treated as a continuous variable. The tests of difference between two successive surveys were performed using survey period as a discrete variable with appropriate contrast matrices. For convenience, the RRs for the 5-year cycles were re-expressed as the equivalent RR for a 1-year change.

Differences by area and age were tested in the sex-specific GLM. Interaction terms were also tested between sex, age group and area, and survey period in these models. The six surveys were collected independently to minimize the possibility of overlap. Therefore, the covariance was set to zero when testing for differences between surveys. Data were analyzed using SAS (Version 9.1; SAS Institute Inc., Cary, NC.USA) statistical software. All analyses included sample weights that accounted for the unequal probabilities of selection, oversampling, and non-response. An α = 0.05 was used to test all statistical hypotheses.

## Results

Data from 107,621 boys and 107,582 girls, and 60,9091 boys and 642,973 girls were used in the analysis of the 2010 and 1985–2005 data, respectively.

For 2010, the overall, age-adjusted overweight and obesity combined prevalence was 19.2% (95% CI, 19.1–19.4%) among children and adolescents aged 7–18 years (Table 1). Among boys, the age-adjusted overweight and obesity combined prevalence was 23.4% (95% CI, 23.2–23.7%) overall. Among girls, the overall, age-adjusted overweight and obesity combined prevalence was 14.5% (95% CI, 14.2–14.7%). Prevalence was 22.6% (95% CI, 22.4–22.9%) in rural areas and was 16.2% (95% CI, 15.9–16.4%) in urban areas. Compared with the other age groups, the 10–12-year age group had a much higher combined prevalence. The results were similar for obesity only. The age-adjusted prevalence was 8.1% (95% CI, 8.0–8.3%). Prevalence of obesity was higher in urban areas (10.1%) than in rural areas (6.4%) and in boys (10.9%) compared with girls (5.1%) (Table 1).

**Table 1:** Prevalence of overweight or obesity for Chinese children and adolescents aged 7–18 years, 2010.

Sex	Age group	All	Urban	Rural
**Overweight and obesity combined (>P80 (1+10%))**				
Both	7–18	19.2 (19.1–19.4)	22.6 (22.4–22.9)	16.2 (15.9–16.4)
	7–9	20.1 (19.8–20.5)	25.1 (24.5–25.6)	16.4 (16.0–16.9)
	10–12	24.9 (24.5–25.2)	29.0 (28.4–29.5)	21.7 (21.2–22.2)
	13–15	18.8 (18.5–19.2)	22.9 (22.4–23.4)	15.6 (15.2–16)
	16–18	14.9 (14.6–15.2)	17.6 (17.2–18.1)	11.5 (11.1–11.8)
Males	7–18	23.4 (23.2–23.7)	28.3 (27.9–28.7)	19.0 (18.7–19.3)
	7–9	23.9 (23.4–24.4)	30.3 (29.5–31.1)	19.2 (18.6–19.9)
	10–12	29.1 (28.5–29.6)	34.7 (33.9–35.5)	24.7 (24.0–25.4)
	13–15	22.0 (21.5–22.5)	27.5 (26.7–28.2)	17.7 (17.1–18.4)
	16–18	20.1 (19.6–20.6)	24.3 (23.6–25.0)	14.7 (14.1–15.3)
Females	7–18	14.5 (14.2–14.7)	16.1 (15.8–16.4)	12.9 (12.6–13.2)
	7–9	15.7 (15.3–16.2)	19.1 (18.5–19.8)	13.1 (12.6–13.7)
	10–12	20.0 (19.6–20.5)	22.4 (21.7–23.1)	18.2 (17.5–18.8)
	13–15	15.3 (14.8–15.7)	17.8 (17.2–18.5)	13.2 (12.6–13.8)
	16–18	9.1 (8.7–9.4)	10.0 (9.5–10.6)	7.8 (7.4–8.3)
**Obesity (>P80 (1+20%))**				
Both	7–18	8.1 (8–8.3)	10.1 (9.9–10.3)	6.4 (6.2–6.5)
	7–9	8.0 (7.8–8.2)	10.5 (10.1–10.8)	6.2 (5.9–6.5)
	10–12	11.0 (10.7–11.2)	13.3 (12.9–13.7)	9.1 (8.8–9.5)
	13–15	8.2 (8.0–8.5)	10.7 (10.3–11.1)	6.2 (6.0–6.5)
	16–18	6.2 (6.0–6.4)	7.8 (7.5–8.1)	4.1 (3.9–4.3)
Males	7–18	10.9 (10.7–11)	13.8 (13.5–14.1)	8.2 (8–8.4)
	7–9	10.4 (10–10.7)	13.9 (13.3–14.5)	7.7 (7.3–8.2)
	10–12	14.2 (13.8–14.6)	17.5 (16.8–18.1)	11.7 (11.2–12.3)
	13–15	10.6 (10.2–10.9)	14.0 (13.4–14.6)	7.8 (7.4–8.3)
	16–18	9.0 (8.7–9.4)	11.5 (11.0–12.1)	5.8 (5.4–6.2)
Females	7–18	5.1 (4.9–5.2)	6.0 (5.8–6.2)	4.2 (4.1–4.4)
	7–9	5.3 (5.0–5.6)	6.5 (6.1–6.9)	4.4 (4.0–4.7)
	10–12	7.2 (6.9–7.5)	8.6 (8.1–9.1)	6.1 (5.7–6.5)
	13–15	5.6 (5.3–5.9)	7.0 (6.6–7.4)	4.4 (4.1–4.8)
	16–18	3.0 (2.8–3.2)	3.5 (3.2–3.8)	2.3 (2–2.5)

During the 1985–2010 period, the risk of being obese was significantly higher for urban males (RR, 2.11; 95% CI, 2.07–2.15) and females (RR, 1.76; 95% CI, 1.71–1.82) compared with rural males and females, after controlling for age and survey period (Table 2). Adolescents aged 16–18 years had a lower risk of obesity (males: RR, 0.86; 95% CI, 0.84–0.89; females: RR, 0.64; 95% CI, 0.61–0.67) compared with children aged 7–9 years, after adjusting for survey period and residence area. However, the risk of obesity was significantly higher for adolescents 10–12 years compared with children aged 7–9 years (males: RR, 1.40; 95% CI, 1.37–1.44; females: RR, 1.48; 95% CI, 1.43–1.54). Similarly, obesity or overweight was more likely to occur among children or adolescents who were males, urban, and aged 10–12 years.

**Table 2:** Rate ratios for obesity or overweight and obesity combined prevalence, 1985–2010, RR (95% CI).

Parameter	All	Male	Female
Obesity, >P80 (1+20%)			
Males/Females	1.93 (1.90–1.97)		
Survey period	1.55 (1.54–1.56)	1.59 (1.58–1.60)	1.49 (1.48–1.50)
Urban/Rural	1.99 (1.95–2.02)	2.11 (2.07–2.15)	1.76 (1.71–1.82)
Age group, y			
10–12/7–9	1.43 (1.40–1.46)	1.40 (1.37–1.44)	1.48 (1.43–1.54)
13–15/7–9	1.12 (1.10–1.15)	1.09 (1.05–1.12)	1.20 (1.16–1.25)
16–18/7–9	0.79 (0.77–0.81)	0.86 (0.84–0.89)	0.64 (0.61–0.67)
Overweight and obesity combined, >P80 (1+10%)			
Males/Females	1.44 (1.42–1.45)		
Survey period	1.41 (1.41–1.42)	1.46 (1.46–1.47)	1.35 (1.34–1.35)
Urban/Rural	1.57 (1.55–1.58)	1.67 (1.65–1.69)	1.42 (1.40–1.44)
Age group, y			
10–12/7–9	1.29 (1.27–1.30)	1.25 (1.23–1.27)	1.35 (1.32–1.37)
13–15/7–9	1.04 (1.02–1.05)	0.97 (0.95–0.99)	1.14 (1.12–1.17)
16–18/7–9	0.81 (0.80–0.82)	0.87 (0.85–0.88)	0.73 (0.72–0.75)

The results of trend analyses for the 25-year period revealed a significant trend in obesity prevalence during 1985–2010 in males (RR, 1.59; 95% CI, 1.58–1.60, Table 2) and in females (RR, 1.49; 95% CI, 1.48–1.50). The annual increase in the ratios of obesity prevalence was 1.10 for males and was 1.08 for females (Table 3). Results of trend tests that used survey period as a discrete variable were consistent with results of trend tests that used survey period as a continuous variable. In 2010, the prevalence of obesity, and of overweight and obesity combined, among children or adolescents were significantly higher than at the previous five time points.

**Table 3:** Estimated annual increase in the rate ratios of obesity and overweight prevalence during 1985–2010 and 2005–2010, in different subpopulations, RR (95% CI).

Subpopulation	Overweight and obesity		Obesity	
	1985–2010	2005–2010	1985–2010	2005–2010
Males	1.08 (1.08–1.08)	1.04 (1.04–1.04)	1.10 (1.10–1.10)	1.05 (1.04–1.05)
Males, urban	1.09 (1.08–1.09)	1.02 (1.02–1.03)	1.10 (1.10–1.10)	1.03 (1.02–1.03)
Males, rural	1.07 (1.07–1.07)	1.07 (1.06–1.07)	1.10 (1.10–1.10)	1.09 (1.08–1.10)
Females	1.06 (1.06–1.06)	1.04 (1.03–1.04)	1.08 (1.08–1.08)	1.04 (1.03–1.05)
Females, urban	1.07 (1.06–1.07)	1.02 (1.02–1.03)	1.08 (1.08–1.09)	1.02 (1.01–1.03)
Females, rural	1.06 (1.06–1.06)	1.05 (1.05–1.06)	1.08 (1.08–1.09)	1.04 (1.03–1.05)
All	1.07 (1.07–1.07)	1.04 (1.03–1.04)	1.09 (1.09–1.09)	1.04 (1.04–1.05)

**Figure 1:**
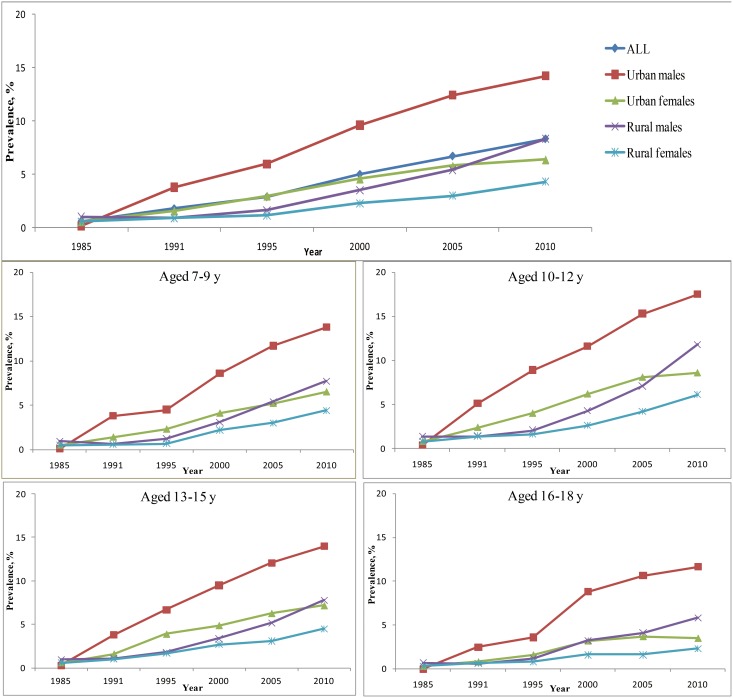
Prevalence of obesity in Chinese males and females aged 7–18 years.

**Figure 2:**
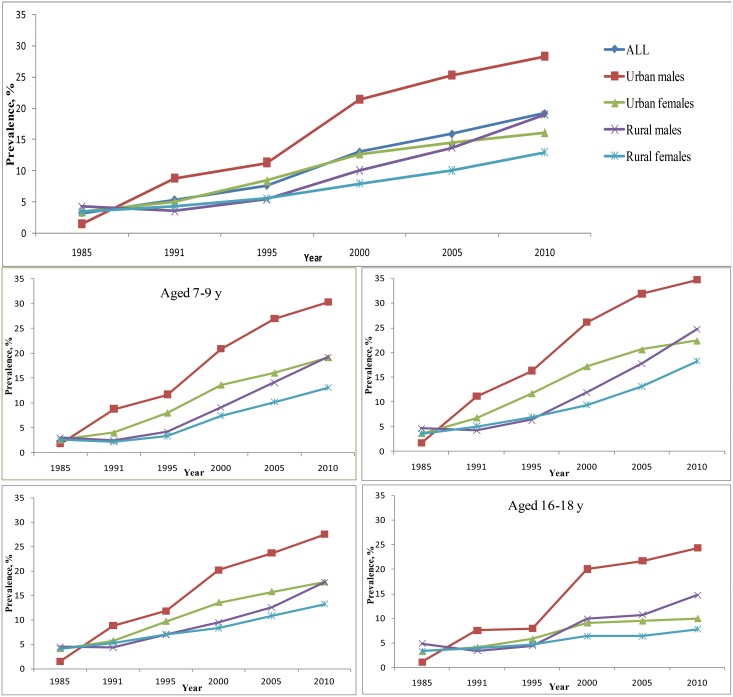
Prevalence of overweight and obesity combined for Chinese males and females aged 7–18 years.

During 1991–2010, the prevalence of obesity or overweight among males from urban areas was continuously higher compared with other subpopulations (Figures 1, 2). Compared with children from urban areas, the obese or overweight prevalence for children from rural areas had a more obvious increasing trend during 2005–2010. This pattern was especially true for males from rural areas. The annual increase in RR was 1.07 (95% CI, 1.06–1.07, Table 3) for overweight or obesity and was 1.09 (95% CI, 1.08–1.10) for obesity. The rate of increase was slower for girls from urban areas.

## Discussion

The results from the analysis of the CNSSCH data provided estimates of the prevalence of children and adolescent obesity in China and of the increasing trends in obesity over the past 25 years. In 2010, 8.1% of Chinese children and adolescents (aged 7–18 years) were obese and 11.1% were overweight. For the 1985–2010 period, trends in obesity and overweight prevalence among children and adolescents were significantly increasing for the total population and for the sex, age, and area subpopulations, and the pace of obesity epidemic among boys in urban was much faster than that in other subgroups. During 2005–2010, the prevalence in obesity in boys from rural areas increased rapidly, at approximately 9% per year.

We found that the prevalence of overweight and obesity among Chinese children continues to increase. This trend has been increasing at a high rate since the early 1990s, and the values for the incremental increases are exceptionally high. Other industrialized and developing countries are also experiencing this increasing trend in childhood obesity. It is accompanied by a significant shift from undernutrition towards overnutrition. These dramatic shifts may be the result of improvements in diet and reduction in physical activity [8], [15]–[19]. Over the past two decades, the per capita gross domestic product in China has increased by more than 10-fold [20]. This shift in economic welfare has been accompanied by increases in food availability and dietary intake, particularly of nutrient- and energy-dense diets. Large proportions of the subpopulations in China have benefited from these improved economic and social conditions.

A large component of obesity is clearly due to the decrease in physical activity and reduced energy expenditure during transportation. More leisure time is available, which is often spent as inactive leisure time [21]–[23]. The abundance of enticements lead to reduced physical activity, such as watching television and playing computer games. Since 1990, television ownership has increased remarkably in China. Television viewing besides reducing physical activity also leads to increased consumption of energy-rich foods through incessant commercial advertisement. Television watching has been closely related to childhood obesity [8], [24]–[26]. Moreover about 30% of Chinese boys and 15% of Chinese girls spent >2 h per day playing computer games [10]. This situation will become worse due to the popularity of smart phones. Inactivity also increased with the establishment of the college entrance examination system, because more time was needed for school and homework. The specific reasons for the imbalance between dietary intake and energy expenditure in different regions need further study.

We reported a decreasing trend in the growing speed in the obesity among Chinese children except rural boys. The study suggest that the increasing prevalence of obesity previously observed may not continue at a similar rate. In fact, the increasing prevalence appeared to be retarded. However, our study did not suggest that the prevalence of obesity is declining in any group. Currently, little is known about the exact causes of the observed trends in body weight. Childhood obesity continues to increase in developing countries while it has apparently plateaued or slightly growth in developed countries [27]–[29]. China is a developing country with rapid growth in economy. The health care system in China is significantly improved and Chinese people may have a better knowledge of health, resulting in a lower speed in the increasing of obesity.

Analysis of the six CNSSCH surveys revealed that rates of obesity or overweight were always higher in urban areas compared with rural areas. Urban residents have been the first to experience the benefits from China’s economic development. They have experienced dramatic lifestyle changes in the past two decades. For children, family income and urbanization may be linked to dietary factors (e.g., the consumption of energy-dense foods instead of fruits and vegetables), and to factors associated with sedentary behavior (e.g., increased television watching, and the absence of safe streets, parks, and play areas) [30]. In general, urban residents experience better living conditions and participate in less physical activity than rural residents [31], [32]. Results of studies of the links between obesity and family socioeconomic status suggest that excess weight gain among children is more prevalent among higher income families in less industrialized societies, especially as they move to urban areas [15]. Rural residents have also experienced dramatic lifestyle changes in the past several years. The changes experienced by urban children are also being experienced by rural children, which may explain why the prevalence in obese or overweight increased at a greater rate in rural children than in urban children during 2005–2010.

Sex group differences in overweight and obesity prevalence among children and adolescents have continued over time. These differences are also present among different areas and among different age groups [5], [12]. The prevalence was higher in girls than boys in many Western countries [33], [34]. However, the prevalence of childhood obesity was higher in boys than girls in China. They may be related to China’s one-child policy, and the society’s traditional preference for sons, particularly in rural areas [15], [35]. Boys are likely to enjoy more of the family’s resources and to be better raised than girls, therefore they might obtain more food and participate in less work. Moreover Chinese boys generally have different self-concept of body image compared with Western boys, and in accordance with the Chinese cultural value, the obesity in boys are not recognized as detrimental or unbearable [36]. Parents and grandparents also hope that their boy is chubby and their girl is as pretty as Snow White. So, they give boy more food than girl. The sex difference in overweight trends may also be related to the emerging body image (preference for thinness) among Chinese adolescent females [15], [37], [38]. The lifestyle changes may have also contributed to the gender disparity in the prevalence of obesity. Chinese boys had soft-drinks more frequently, fewer tried to lose weight by restricting diet and spent more time playing computer games than girls [39]. More fat during childhood is associated with an increased risk of heart disease in adulthood [2]. Epidemics of chronic diseases among Chinese males are likely to occur in the future.

Because different definitions of obesity in children and adolescents are used throughout the world, it can be difficult to compare estimates in China with estimates in other countries. Although cut-offs for BMI are frequently used in definitions of obesity (e.g., the NCHS (National Center for Health Statistics) and the IOTF (International Obesity Task Force) sex- and age-specific BMI references), BMI does not seem to be a suitable measure for the growth patterns of Chinese juveniles [40], [41]. In 2010, mean BMI values among American children and adolescents aged 6–11 years and 12–19 years were 18.3 and 23.8 for boys, and 18.5 and 23.6 for girls, respectively. They were 17.1 and 19.9 for Chinese boys and 16.3 and 19.6 for Chinese girls for the same age groups [29]. The level of obesity among Chinese adolescents is lower than it is in American adolescents. However, China will soon confront issues similar to the United States.

Our study had the following limitations. First, obesity was defined based on weight-for-height, which is an imperfect measure of body fat. However, weight-for-height is highly correlated with body fat at the higher values for weight-for-height. This study explored relatives change in prevalence of obesity and, regardless of the measure used, a trend was apparent. Furthermore, we used a statistical definition of obesity that was based on a comparison to the 1985 reference population represented in the CNSSCH growth charts. The charts were created for comparison within specific sex and age groups and not for comparison across sex and age groups. Sex or age differences in prevalence in 2010 may reflect differences from the original reference population. A second limitation was that the study population was comprised only of people of the Han nationality and did include ethnic minorities. However, the Han nationality accounts for 92.7% of China’s population. We will investigate prevalence and trends in overweight among China’s minority children in our next study. Finally, this study used data from six cross-sectional surveys, as each was conducted on different subjects. It is possible that unintentional errors occurred when estimating the prevalence of overweight and obesity and comparing the trends. However, the prevalence estimated in each CNSSCH was standardized according to the age distribution of 2010 population for the purpose of comparison. Despite these limitations, our findings make a significant contribution that addresses the health characteristics of Chinese children and adolescents.

In summary, our results reveal that the increasing trend in the rate of overweight and obesity in Chinese children is continuing. The prognosis is worrisome. A dramatic and steady increasing trend was witnessed among all sex and age subgroups from urban and rural areas. The incremental increases among rural boys were exceptionally high during 2005–2010. Although China is at the early stage of an obesity epidemic, the prevalence of overweight or obesity in the urban population has reached the same level as some developed countries. These trends present many critical public health challenges. Investments promoting school health and infrastructure that are designed to stop and to reverse the trend from physically active to sedentary pastimes are the most effective weapons available to policymakers. Public health policy should specifically address these issues.
